# Effect of Fluoxetine and Acacetin on Central Vestibular Compensation in an Animal Model of Unilateral Peripheral Vestibulopathy

**DOI:** 10.3390/biomedicines10092097

**Published:** 2022-08-27

**Authors:** Bérénice Hatat, Romain Boularand, Claire Bringuier, Nicolas Chanut, Christian Chabbert, Brahim Tighilet

**Affiliations:** 1 Vertidiag, 34080 Montpellier, France; 2 Aix Marseille Université-CNRS, Laboratoire de Neurosciences Cognitives, LNC UMR 7291, 13331 Marseille, France; 3 GDR Physiopathologie Vestibulaire, Unité GDR2074, CNRS, 13003 Marseille, France

**Keywords:** vestibular disorders, vestibular compensation, vertigo, vestibular function recovery, fluoxetine, acacetin

## Abstract

Damage to the peripheral vestibular system is known to generate a syndrome characterized by postural, locomotor, oculomotor, perceptual and cognitive deficits. Current pharmacological therapeutic solutions for these pathologies lack specificity and efficacy. Recently, we demonstrated that apamin, a specific SK channel blocker, significantly reduced posturo-locomotor and oculomotor deficits in the cat and the rat. The aim of the present study was to test the antivertigo potential of compounds belonging to the SK antagonists family, such as Acacetin and Fluoxetine. Young rats were subjected to unilateral ototoxic lesions of the vestibular organ using transtympanic administration of arsanilic acid (TTA) to evoke unilateral vestibular loss (UVL). Vestibular syndrome was monitored using behavioural evaluation allowing appreciation of the evolution of static and dynamic posturo-locomotor deficits. A significant effect of the TTA insult was only found on the distance moved, the mean body velocity and the not moving time. From day 2 to week 2 after TTA, the distance moved and the mean body velocity were significantly decreased, while the not moving time was significantly increased. Acacetin does not evoke any significant change in the vestibular posturo-locomotor parameters’ kinetics. Administration of Fluoxetine two weeks before TTA and over three weeks after TTA (preventive group) does not evoke any significant change in the vestibular posturo-locomotor parameters’ kinetics. Administration of Fluoxetine from three weeks after TTA significantly delayed the functional recovery. This study demonstrates that Acacetin or Fluoxetine in TTA vestibulo-injured rats does not bring any significant benefit on the posture and locomotor balance deficits.

## 1. Introduction

Peripheral vestibulopathies represent 5% of all medical prescriptions in people over 50 [1]. Unilateral damage to vestibular sensory sensors induces a characteristic acute vestibular syndrome in various species, including humans. This syndrome is the result of altered vestibulo-spinal, vestibulo-oculomotor, vestibulo-vegetative reflexes, as well as modification of vestibulo-cortical signals [2]. It consists of a postural imbalance at rest and during movement, a loss of coordination of eye movement in orbit, associated with perceptual, cognitive and neurovegetative disorders. This characteristic phenotype is also observed during unilateral peripheral vestibular involvement due to vestibular neuritis, Meniere’s disease or a labyrinthine fistula [2]. Pharmacological approaches to attenuate the acute vertigo episodes remain poorly effective due to the lack of specific treatments available for these pathologies [3,4,5]. 

SK channels (small conductance Ca^2+^-Sensitive K^+^ channel) are widely expressed in the central nervous system [6] and especially in the vestibular nuclei [7]. A recent study demonstrated that unilateral vestibular neurectomy (UVN) induces both ipsi- and contralateral up-regulation of the apamin-sensitive-calcium-activated small conductance K^+^ (SK) channels, over the first days following the insult [8]. It was also demonstrated that apamin administration, a specific blocker of the SK channels, during the acute phase of the vestibular syndrome, significantly reduced both the posturo-locomotor and vestibulo-ocular deficits induced by the UVN in cats [8]. We recently demonstrated that the antivertigo (AV) effect of apamin is also found in a UVN rodent model, and that other SK antagonists produce a trend of AV effect when administrated during the acute phase of the vertigo syndrome [9]. Conversely, the vertigo syndrome is worsened upon administration of the SK channel agonist. Taken together, these data reinforce SK channels as a pharmacological target for modulating the manifestation of the vertigo syndrome during acute peripheral vestibulopathies.

Several ligands of SK channels have been identified, including Fluoxetine and Acacetin (see [10] for review). Fluoxetine, is also a selective serotonin reuptake inhibitor (SSRI). It is commonly prescribed clinically as Prozac^®^ to treat emotional disorders by inhibiting serotonin reuptake. The re-use of drugs in pathologies where the therapeutic options are weak, as is the case of vestibular pathology, can be beneficial as demonstrated with the re-use of trifluoroperazine in models of traumatic brain and spinal cord injuries [11,12]. A comorbidity between emotional disorders and vestibular dysfunction has been established in the literature [13]. However, there are very few studies in the literature that mention the use of SSRI in patients with vestibular dysfunction [14]. The few studies currently available only concern the use of SSRI in Persistent Postural-Perceptual Dizziness (PPPD) [15]. Fluoxetine has a good affinity for SK channels and acts as a channel inhibitor [16]. Its role in the control of posture and balance has also been reported in humans and animal models [17,18]. Acacetin, a natural flavonoid, possesses a broad spectrum of pharmacological and biochemical activities, such as neuroprotection, antiinflammation, inhibition of monoamine oxidase and, also, antidepressant-like efficacy as demonstrated with Fluoxetine [19]. This project aims at assessing for the first time in rodents the potential of Fluoxetine and Acacetin of modulating the posturo-locomotor deficits evoked following ototoxic peripheral unilateral vestibular insults.

## 2. Materials and Methods

### 2.1. General Procedures

All experiments are performed in accordance with the National Institutes of Health’s Guide for Care and Use of Laboratory Animals (NIH Publication no. 80-23) revised in 1996 for the UK Animals (Scientific Procedures) Act of 1986 and associated guidelines or the Policy on Ethics approved by the Society for Neuroscience in November 1989 and amended in November 1993 and under the veterinary and National Ethical Committee supervision (French Agriculture Ministry Authorization n°F34-172-05). The present study was specifically approved by the ethics committee MP-CEPAL n°22 from the French National Committee of animal experimentation. Male Long Evans rats (7–8 weeks, Janvier, France) were housed in groups of five under constant temperature (20 ± 2 °C), humidity (55 ± 5%) and brightness conditions (lower than 110 Lux). Rats were housed under a 12 h–12 h diurnal light variation (lights on from 07:00 to 19:00 h) with food and water freely available. Handling (~5 min per animal per day) was performed one week before the beginning of the tests.

### 2.2. Treatment

Four groups of rats received unilateral transtympanic injections of 100 μL of arsanilic acid (TTA) at a concentration of 50 mg/mL in the left ear. I.p. injections were made as follows:

- TTA–Saline (*n* = 14): daily i.p. injections of Saline (0.9% NaCl in water) over the four days following the vestibular insult.
- TTA–Acacetin (*n* = 14): daily i.p. injections of Acacetin (25 mg/kg) over the four days following the vestibular insult.
- TTA–Fluoxetine (*n* = 12): daily i.p. injections of Fluoxetine (10 mg/kg) from the day of the surgery to the end of the third week (total of 22 injections)
- TTA–Fluoxetine preventive (*n* = 12): daily i.p. injections of Fluoxetine (10 mg/kg) two weeks before the surgery until the end of the third week after surgery (total of 36 injections).

I.p. injections were all made in the morning before experiments. When surgery was planned the same day, i.p. injections were made during the surgery when animals were under anaesthesia. The choice of the time window of administration of Fluoxetine was made to highlight an action of Fluoxetine on the SK channels (acute window of 4 days) but also an action on serotonin transmission (chronic window of 3 and 5 weeks). Acacetin was administrated in acute condition to check its action on SK channels.

### 2.3. Arsanilic Acid Lesioning Procedure

Each rat received a single unilateral dose (50 mg/mL–0.1 mL per ear) of arsanilic acid (Sigma-Aldrich-CAS: 98-50-0) dissolved in 0.9% saline solution in the left ear. Animals were under volatile anaesthesia (2% Isoflurane in oxygen (flow rate of 2 L/min)). The drug was injected through the anterior part of the tympanum using a 1 mL syringe (needle diameter, 0.8 mm) and arsanilic acid was deposited into the middle-left ear cavity. The control group received a single unilateral transtympanic injection of 0.9% saline solution (0.1 mL) in the left ear. 

### 2.4. Scoring of Vestibular Syndrome

Unilateral syndromes were quantified during the open field video tracking at different time points: 24 h, 48 h, 72 h, 1 week, 2 weeks and 3 weeks after the transtympanic injection of arsanilic acid. The vestibular syndrome was first stimulated by elevating the rat by the tail about twenty centimetres from the support in order to exacerbate the expression of symptoms. The vestibular syndrome was then assessed for 2 min according to a previously published vestibular scale used for all vestibular insults [20]. A score of 0 (no symptoms) to 3 (severe symptom) was assigned to the following 11 criteria, for a maximum total score of 33: torsional movement of the body when the rat is lifted by the tail over the support (tail hanging behaviour), landing reflex of the rat when dropped on the support (landing reflex), prostration time (immobility), ability to stand up on the hind legs (rearing), quality of gait and general movements of the animal (quality of displacement), body height and lift (body height), lateral tilt of the head (head tilt), reverse walk (retropulsion), concentric trajectories (circling), vertical repetitive movements of the head (bobbing) and general condition.

### 2.5. Support Surface

Support surface is a sensitive parameter measured by the surface delimited by the four paws of the animal after a tail hanging landing test. This test consisted in taking the animal by the tail and lifting it vertically over a height of about 50 cm (lift duration 2 s; position holding at upper position: 1 s). To quantify the support surface, animals were placed in a device with a graduated transparent floor that allowed them to be filmed from underneath. A scale drawn on the bottom served to take measurements of the four paws’ locations. When the animal landed after the tail hanging test and touched the ground, we took a capture of the four paws’ locations. Between 10 and 15 measurements were taken for each rat at each time point during recovery (24 h, 48 h, 72 h, 1 week, 2 weeks and 3 weeks after the unilateral lesion). An average was calculated for each time point. The support surface was measured using an image analysis system called GNU Octave.

### 2.6. Open Field and Video Tracking

The open field apparatus was an inescapable square area (80 × 80 × 40 cm) without bedding litter. Animals were placed in the centre of the field, considered more anxiogenic compared to the periphery, and their behaviour was recorded for 5 min using a digital camera with Ethovision^TM^ XT 15 software (Noldus) which automatically detected the following body-points throughout the recordings: nose-point, centre point and tail base. This test was carried out before the unilateral lesion (time point called pre-op), 24 h, 48 h, 72 h, 1 week, 2 weeks and 3 weeks after the transtympanic injection of arsanilic acid. Three days before the pre-op recording, each animal was allowed to freely explore the maze for 2 min. Light was fixed at 40–45 Lux at the centre and 30–35 Lux in each corner of the maze. The surfaces of the open field were cleaned thoroughly between animals with an Ethanol solution (20%). We used one profile for five variables (distance moved, mean body velocity, max body velocity, meander and not moving time) we selected for analysis. This profile, a tool included in EthoVision XT software, uses the minimal distance moved (MDM) smoothing method to filter out small movements (<0.7 cm) of the animal’s centre point that are caused by random noise. “Duration not moving” of animals was calculated with an average interval of three samples, one sample is generated every 0.04 s during the whole time of the video and a threshold of 2.00 cm/s for start and 1.75 cm/s for stop velocity. Mean body velocity and max body velocity were calculated with an average of three samples.

### 2.7. Statistical Analyses

Statistical analyses were performed under the supervision of a statistician expert. Results are expressed as means ± standard error of mean (SEM). Two-way analysis of variance (ANOVA) and repeated measures (RM-ANOVAs) were used to analyse each parameter of open-field, scoring and support surface. When ANOVA indicated a significant overall effect, post hoc analysis was performed using Tukey’s test. A significant difference is indicated by * if *p* < 0.05, ** if *p* < 0.01, *** if *p* < 0.001 and **** if *p* < 0.0001.

## 3. Results

### Effect of Fluoxetine and Acacetin on the Ototoxically-Induced Vestibular Syndrome

**Figure 1:**
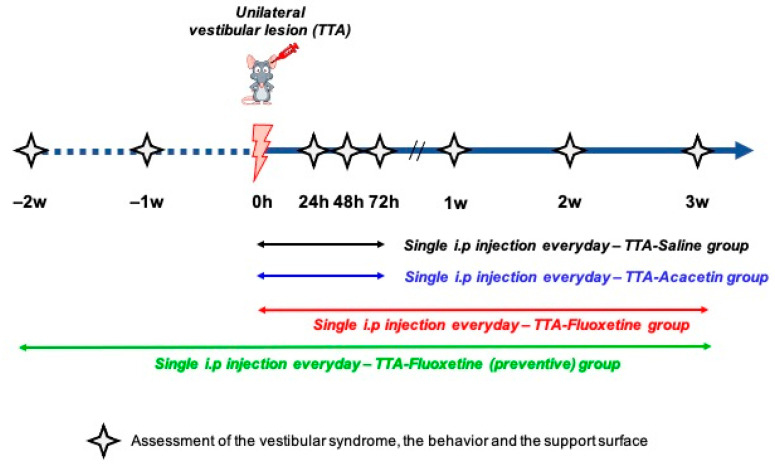
Study timeline of Fluoxetine, Acacetin and Saline groups. Assessment of central vestibular compensation was made through the analysis of the vestibular syndrome, behaviour and support surface. Each analysis was carried out at several time points: before the transtympanic injection (referred as pre-op), 24 h, 48 h, 72 h, 1 week, 2 weeks and 3 weeks after unilateral transtympanic injection of arsanilic acid.

Four groups: TTA–Saline (*n* = 14), TTA–Acacetin (*n* = 14), TTA–Fluoxetine preventive (*n* = 12) and TTA–Fluoxetine (*n* = 12) received unilateral transtympanic injections of arsanilic acid at a concentration of 50 mg/mL (100 μL per ear). TTA–Saline and TTA–Acacetin groups received four i.p. injections of saline (0.9% of NaCl in water) or Acacetin (25 mg/kg). The first i.p. injection was made during anaesthesia necessary for the transtympanic injection and then 24 h, 48 h and 72 h after the vestibular insult. TTA–Fluoxetine preventive group was daily intraperitoneally administrated with Fluoxetine during two weeks before the vestibular insult and three weeks after. TTA–Fluoxetine group was daily administrated intraperitoneally with Fluoxetine over three weeks after the vestibular insult. Assessment of central vestibular compensation was made through analysis of the vestibular syndrome, behaviour in the open-field and support surface. Each analysis was carried out at several time points: before the transtympanic injection (referred as pre-op), 24 h (D1), 48 h (D2), 72 h (D3), 1 week (W1), 2 weeks (W2) and 3 weeks (W3) after unilateral transtympanic injection (see below study plan—Figure 1).

**Figure 2:**
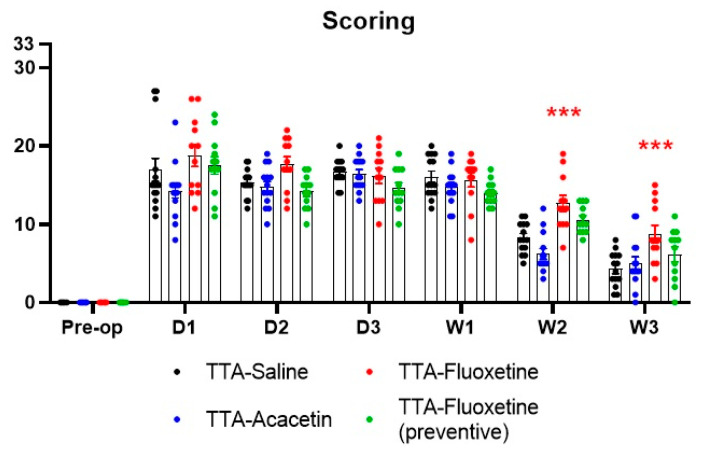
Time course of the vestibular syndrome evaluated through behavioural subjective scoring. Data represent mean ± SEM; statistically significant differences (*** *p* < 0.001) compared to control group, two-way ANOVA applies to all graphs. Significant differences between TTA–Saline and TTA–Fluoxetine groups are indicated by red stars.

The time course of the vestibular syndrome observed in the four TTA groups was similar as those previously reported by Vignaux and colleagues [21]. The vestibular syndrome was maximum 24 h after UVL (D1) and remained high the days following the vestibular insult. It then progressively decreased in all groups from week 2 (W2) to week 3 (W3) (Figure 2). No significant difference between each group and the control group (TTA–Saline) was observed except at W2 and W3 for the TTA–Fluoxetine group. Indeed, it seems that administration of Fluoxetine after the vestibular insult worsened the general behaviour of these animals, since their scoring statistically differed from the control group at these two time points. This difference was not observed with the group administrated with Fluoxetine two weeks before UVL (TTA–Fluoxetine preventive) or with Acacetin.

**Figure 3:**
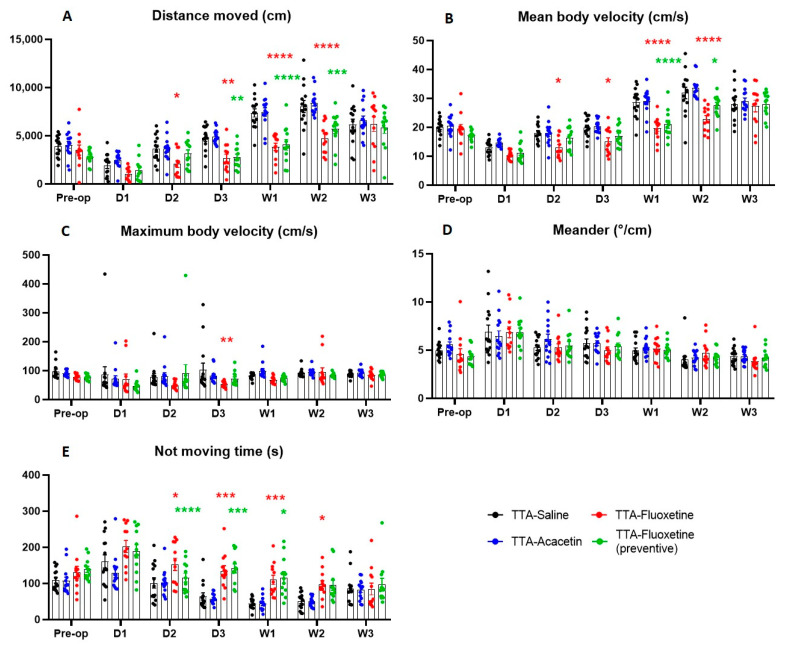
Illustration of the average distance moved (**A**), mean body velocity (**B**), maximum body velocity (**C**), meander (**D**) and not moving time (**E**) automatically measured in a 10 min-session in the open-field. Data represent mean ± SEM; statistically significant differences (* *p* < 0.05; ** *p* < 0.01; *** *p* < 0.001; **** *p* < 0.0001) compared to the control group, two-way ANOVA applies to all graphs. Significant differences between TTA–Saline and TTA–Acacetin groups, TTA–Saline and TTA–Fluoxetine (preventive) groups and TTA–Saline and TTA–Fluoxetine groups are indicated by blue, green and red stars respectively.

Assessment of the distance moved by animals in the open field demonstrated that this parameter did not significantly vary at any time points between the control group (TTA–Saline) and Acacetin group (Figure 3A). Thus, injections of Acacetin did not have any effect on the distance moved. Nevertheless, the distance moved was statistically different between the control group and the two Fluoxetine groups. Administration of Fluoxetine significantly reduced the distance moved from D2 to W2 after UVL for the non-preventive group (TTA–Fluoxetine) and from D3 to W2 after UVL for the preventive group (TTA–Fluoxetine preventive).

Injections of Acacetin did not have any effect on the mean body velocity either. This parameter did not significantly vary between TTA–Saline and TTA–Acacetin groups at any time point (Figure 3B). Nevertheless, this parameter was affected by the administration of Fluoxetine. The mean body velocity was statistically reduced from D2 to W2 after UVL for the non-preventive group (TTA–Fluoxetine) and from W1 to W2 for the preventive group (TTA–Fluoxetine preventive). At W3, the mean body velocity of each group was approximatively the same and increased relative to their pre-op value (data not shown). 

Assessment of the maximum body velocity demonstrated that this parameter did not significantly vary between the control group (TTA–Saline) and the three other groups except at D3 after UVL for the group TTA–Fluoxetine (Figure 3C). 

No significant effect was either observed between the control group and the three other groups on the meander parameter (Figure 3D). However, animals from groups TTA–Fluoxetine and TTA–Fluoxetine (preventive) tended to display a higher deviation of the walk relative to their pre-op value compared to those receiving Acacetin, although this tendency was never statistically significant, whatever the time point considered.

Automatized evaluation demonstrated that not moving time did not significantly vary between the control group (TTA–Saline) and Acacetin group (Figure 3E). Injections of Acacetin did not have any effect on the not moving time. However, administration of Fluoxetine significantly increased this parameter from D2 to W2 after UVL for the non-preventive group (TTA–Fluoxetine) and from D3 to W2 after UVL for the preventive group (TTA–Fluoxetine preventive). 

**Figure 4:**
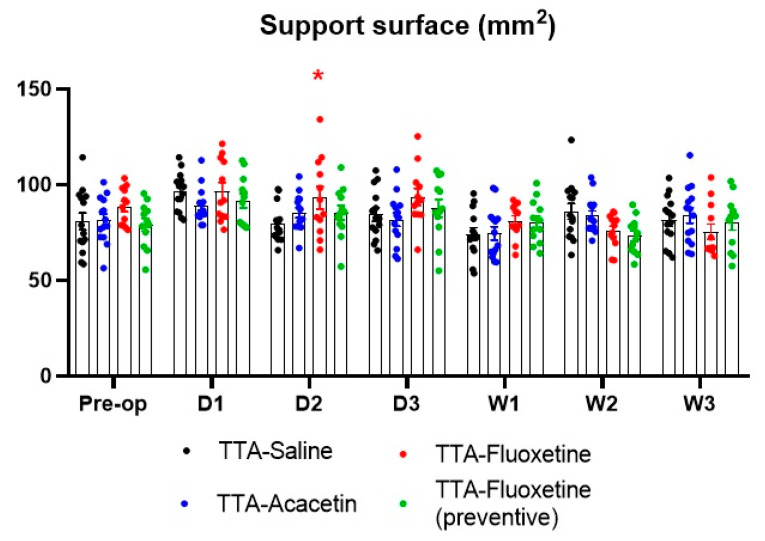
Illustration of the calculated support surface area. Data represent mean ± SEM; statistically significant differences (* *p* < 0.05) compared to the control group, two-way ANOVA applies. Significant differences between TTA–Saline and TTA–Acacetin groups, TTA–Saline and TTA–Fluoxetine (preventive) groups and TTA–Saline and TTA–Fluoxetine groups are indicated by blue, green and red stars, respectively.

Support surface measurements did not significantly differ between the control group and the three other groups except at D2 after UVL for the TTA–Fluoxetine group regarding Figure 4. 

**Figure 5:**
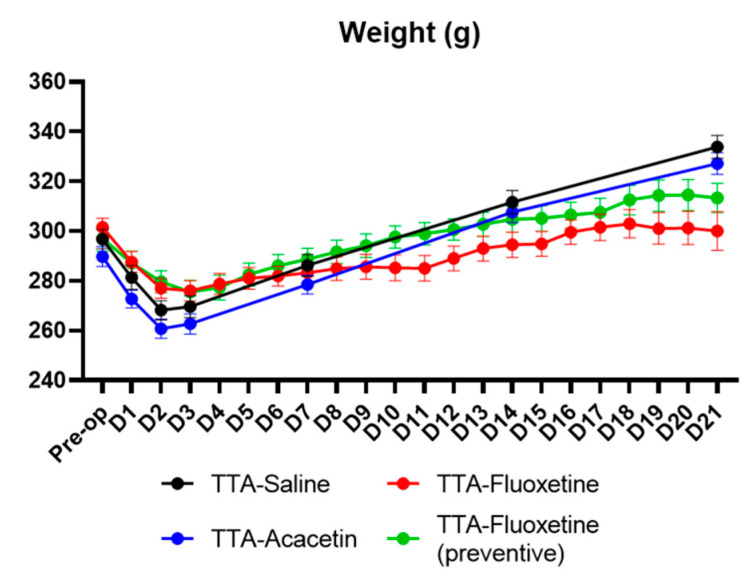
Illustration of the weight monitoring of each group at several time points after UVL.

Body weight was monitored in all groups at each i.p. injection or time point of the study. Animals from TTA–Fluoxetine and TTA–Fluoxetine (preventive) groups did not gain weight or gained very little over the weeks after UVL, whereas animals from TTA–Acacetin and TTA–Saline groups gained on average 12–13% extra weight between the pre-op and W3 (Figure 5).

## 4. Discussion

### 4.1. Irreversible Unilateral Peripheral Vestibular Deafferentation Induced by Transtympanic Administration of Arsanilic Acid: A Model Comparable to the Unilateral Vestibular Neurectomy (UVN)

The posturo-locomotor deficits induced by the transtympanic administration of arsanilic acid (TTA) follows a specific kinetic. In the acute phase of the syndrome (D1), the distance moved and the mean body velocity are decreased, whereas the not moving time and the meander are increased reflecting the state of dizziness of animals. During the compensation phase, from D2 to W3, the distance moved and the mean body velocity are gradually increasing, surpassing even the pre-op values. On the other hand, the meander and the time of immobility decrease gradually until reaching values even lower than those obtained at the preoperative time. The expression kinetics of these parameters is roughly similar to those obtained in the case of unilateral vestibular neurectomy (UVN). In a recent study, the distance moved, mean body velocity, maximum body velocity, meander and not moving time have also been assessed in the open field paradigm in the UVN model [22]. It was observed that the amplitude of the symptoms affecting these parameters was significantly more severe than in the TTA model. As an example, the alteration of the different parameters in the acute phase at D1 is much more accentuated after UVN than after TTA administration. This difference in the vestibular syndrome severity encountered between the two models may rely on the different nature of the peripheral vestibular deafferentation. UVN is achieved through surgical section of the vestibular nerve between the Scarpa’s ganglion and the brainstem. It induces full, irreversible and sudden loss of the inputs arising from both vestibular endorgans and Scarpa ganglion neurons [20]. TTA vestibular damage is also irreversible, but restricted to the vestibular sensory organs, without affecting the Scarpa’s ganglion [21]. In this respect, it resembles the excitotoxic damage induced by glutamate agonist transtympanic (TTK) administration [23], except that the hair cells are damaged in the TTA model conversely to the TTK model and that the insult takes place progressively over several days. In the TTA model, Scarpa’s ganglion neurons continue to feed vestibular nuclei (VN) neurons with afferent tonic input related to resting discharge of their axons [24]. This may support the discrepancies observed in vestibular syndrome between the two models.

### 4.2. Lack of Benefit of Acacetin on the Posturo-Locomotor Component of the TTA–Induced Vestibular Syndrome

Over the several parameters measured in this study, administration of Acacetin did not improve any. Thus, it can be concluded that administration of Acacetin did not bring benefit in alleviating vestibular disorders in the present TTA model and current administration conditions (time window and dosage). It cannot also be excluded that blockade of SK channels by Acacetin had no effect on the vestibular syndrome in the TTA model. Acacetin has been reported to significantly suppress microglial activation in a neuroinflammation mouse model [25]. This suggests that it may act as a potential therapeutic agent for brain diseases involving neuroinflammation. Acacetin also inhibits glutamate release and prevents kainic acid-induced neurotoxicity in rats [26]. Considering these properties, it cannot be excluded that Acacetin may act on the UVN model through inhibition of the strong microglia reaction and inflammation observed in the deafferented VN [27], as well as on the TTK model through inhibition of central glutamate release following vestibular afferent excitation [23]. SK channels are positively regulated in the UVN model. This may explain the beneficial effect of apamin (a pharmacological agent of the SK channel blockers family) on both the cat [8] and rat [9] UVN models. It is, therefore, likely that the TTA model does not induce the same SK channels upregulation in the VN. This will have to be further examined. We have shown that the mechanisms expressed in the deafferented VN depend on the type of vestibular lesion [28]. The more severe the vestibular lesion is, the more robust the reaction mechanisms recruited in the VNs are.

### 4.3. Lack of Benefit of Fluoxetine on the Posturo-Locomotor Component of the TTA–Induced Vestibular Syndrome

Our main results demonstrate that Fluoxetine is deleterious for the vestibular syndrome when administrated preventively or not to the UVL. However, the deleterious effect is less accentuated when administrated preventively. It is well known that Fluoxetine is anorexigenic [29,30,31]. This was confirmed in the present study by difficulty of the Fluoxetine animals to restore their pre-op body weight compared to the Acacetin and control groups. Moreover, among the five parameters measured during the open-field test, three of them (distance moved, mean body velocity and not moving time) were affected by the administration of Fluoxetine. Under the preventive administration of Fluoxetine, the three open-field parameters effects were always delayed by a day or two compared to the non-preventive group. Given the acute mechanism of action of Fluoxetine, one can expect an action of serotonin on the autoreceptors resulting in a decrease in 5HT concentration at the VN level. On the contrary, in a chronic situation, the 5HT autoreceptors are desensitized, and thanks to the inhibition of the reuptake of 5HT by Fluoxetine, an increase in its concentration occurs within the VN. We can hypothesize that an action on the VN SK channels is not privileged in the TTA model, as documented below for Acacetin. A possible reason is that the arsanilic model does not affect the expression of SK channels in deafferented VNs as observed in the UVN model [8,9]. Altering serotonin concentration at the level of the VNs by acute (non-preventive) or subchronic (preventive) treatment with Fluoxetine would probably impact the excitability of the VN neurons through the 5-HT receptors. This may cause a worsening of the syndrome. Serotonin is known to modulate excitability in the VN through its specific receptors [32]. If we intend to draw a parallel with the treatment with Fluoxetine in depression, this treatment is administered in response to the serotoninergic deficit encountered in this pathology [33]. Very few studies have investigated the impact of vestibular injury or stimulation on serotoninergic neurotransmission. Some data show that gravitational [34] and caloric vestibular stimulations [35] are followed by an increased serotoninergic neurotransmission. Other studies attest to an increase in VN serotonin concentration after unilateral labyrinthectomy [36] or unilateral horizontal semi-circular canal occlusion [37]. Consistent with these data, if TTA is thought to cause an increase in VN serotonin levels, this could explain the fact that Fluoxetine treatment disrupts the vestibular syndrome, since this treatment is supposed to raise serotonin levels.

Another possible interpretation of the fact that Fluoxetine treatments disrupts the vestibular syndrome comes from a very fine analysis by Smith’s group [38]. Indeed, dizziness appears to be a common symptom of withdrawal from antidepressants belonging to the selective serotonin reuptake inhibitor (SSRI) category [39,40]. Since the vestibular nucleus complex (VNC) has an abundance of serotonin receptors, the abrupt withdrawal from an SSRI is likely to have a substantial impact on the electrophysiological activity of neurons within it. The authors suggested that the abrupt withdrawal from an SSRI is likely to cause a sudden decrease in serotonin in the VNs, which will disrupt vestibular function causing dizziness [38].

The results collected in this study suggest that Fluoxetine would have a detrimental effect rather than a beneficial one. However, the few data published on Fluoxetine suggest a beneficial effect on the posture [17,18]. It should be noticed that these studies were carried out with a chronic administration of Fluoxetine, which suggests that the observed effects are rather due to a serotoninergic effect (Fluoxetine is a serotonin reuptake inhibitor). An acute or sub-chronic administration of Fluoxetine should, rather, result in an effect via SK channels. Indeed, Fluoxetine produces significant changes on brain serotonin concentrations only after 2 or 3 weeks of chronic treatment. Some vestibular patients who also suffer from anxiety and persistent postural–perceptual dizziness are treated with SSRIs [15,17]. It is likely that these patients present with serotoninergic hypofunction as seen in depressive pathology. Other studies deserve to be deepened to clarify the interaction between serotonin and vestibular pathology in order to better understand why in some patients the emotional component is exacerbated.

### 4.4. Limitations of the Study

I.p. injections are rarely used in clinical practice. However, it has some pharmacokinetic similarities with other routes of administration. In this study, i.p. administrations have been chosen for two main reasons: (i) to avoid useless stress which is, for example, observed with the per os route, and of which it is known that it significantly alters the vestibular compensation; (ii) to be able to compare this present study with our previous studies on the same vestibular pathology model using other i.p.-administered pharmacological compounds [9,27,41].

At this stage, it is difficult to explain the detrimental effect observed with Fluoxetine. Indeed, the concentration used (10 mg/kg) is commonly used in rodents to treat emotional disorders using a chronic administration. It should be noted that Fluoxetine is often administrated orally. Some studies report that a higher dose (more than 20 mg/kg) administrated intraperitoneally can cause death in a few minutes. Some side effects are also observed in patients at the beginning of Prozac treatment. A post-mortem dissection could clarify the potential toxicity of high doses of Fluoxetine. At high doses, some pharmacological compounds may cause an opposite effect to that expected (bell dose–response curve). This could be due to the desensitization or down regulation of some receptors. Chronic treatment of Fluoxetine may also cause desensitization of some serotonin receptor subtypes.

## 5. Conclusions

This study demonstrates a lack of effect of two pharmacological compounds belonging to the SK channel blocker family on the posturo-locomotor component of the vestibular syndrome evoked by an ototoxic insult. This lack of effect is not less interesting because it raises fundamental questions on the pharmacological mechanisms involved in the modulation of the vertigo syndrome and on the therapeutic approach to the management of the vertigo and unstable patient. Indeed, the vestibular syndrome is a mix of both posturo-locomotor, perceptive–cognitive and affectivo-emotional components. Fluoxetine, although one of the SK channel blockers, is also prescribed for its SSRI properties, in the context of the management of anxiety symptoms that accompany vestibular disorders [12]. This work thus raises interesting perspectives, such as the need to better analyze the stress and anxiety component of the vestibular syndrome through tests adapted to animal models of peripheral vestibulopathy. The results of this study on the posturo-locomotor component do not exclude an effect of these compounds on an affective–emotional component (stress, anxiety) of the vestibular syndrome. This is one of the perspectives of the study.

## Data Availability

The data presented in this study are available on request from the corresponding author.
